# Global surgery and the neglected tropical diseases

**DOI:** 10.1371/journal.pntd.0005563

**Published:** 2017-09-28

**Authors:** Vivek Karun, Peter J. Hotez, Todd K. Rosengart

**Affiliations:** 1 School of Medicine, Baylor College of Medicine, Houston, Texas, United States of America; 2 Texas Children’s Hospital Center for Vaccine Development, Departments of Pediatrics and Molecular Virology and Microbiology, National School of Tropical Medicine, Baylor College of Medicine, Houston, Texas, United States of America; 3 James A Baker III Institute for Public Policy, Rice University, Houston, Texas, United States of America; 4 Department of Biology, Baylor University, Waco, Texas, United States of America; 5 Scowcroft Institute of International Affairs, Bush School of Government and Public Service, Texas A&M University, College Station, Texas, United States of America; 6 Department of Surgery, Baylor College of Medicine, Houston, Texas, United States of America; New York Blood Center, UNITED STATES

At the sunset of the Millennium Development Goals there was a global awakening that hundreds of millions of people living in resource-poor countries lacked safe and equitable access to even basic surgeries. In response, a Lancet Commission on Global Surgery was launched in April 2015 under the premise that surgery is an integral component of a properly functioning healthcare system [1]. The commission aims to integrate surgery into the global agenda and identify strategies to provide quality care worldwide in an effort to enhance “universal access to safe, affordable surgical and anesthesia care” [1,2]. The launch coincided with the publishing of their landmark report, which highlighted that an astonishing 5 billion people lack accessible surgical care and that investing in scaling up surgical services until 2030 is necessary and cost effective [2].

Although it is not specifically addressed in the commission’s report, 2 of the largest global surgery programs are already underway in conjunction with mass drug administration (MDA) for the elimination of neglected tropical diseases (NTDs). MDA has served as a cornerstone for programs worldwide aiming to control or eliminate at least 7 of the most common NTDs. Together, these NTDs affect at least 2 billion people who live in extreme poverty, leading to significant physical and mental disability [3]. Furthermore, their impact on health has far-reaching consequences beyond simply the health sector—they also cause profound socioeconomic challenges in the form of lost productivity and income, as well as social inequality [3].

Between 2011 and 2013, out of 1.9 billion people who required annual MDA, over 700 million (37%) received treatment [4]. Through a 2012 London Declaration on NTDs, the major multinational pharmaceutical companies have renewed their commitment to donate the essential medicines required for MDA [5]. According to their fourth progress report, an impressive 1.1 billion treatments were administered to 858 million individuals [5]. Additionally, the global coverage rate exceeded 50% for the first time, as pharmaceutical companies donated drugs to treat 1.5 billion people for NTDs [5]. While the progress of MDA over the past decade has been remarkable in addressing the overall burden of NTDs, it has been limited in its ability to address long-standing comorbidities attributed to the chronic and more severe manifestations of certain NTDs. We bring to attention 2 examples in which surgical care best complements MDA—lymphatic filariasis (LF) and trachoma—and discuss mechanisms to interface these needs with the Lancet Commission on Global Surgery.

## Surgical intervention

As outlined by the commission, the overarching theme remains that basic surgical options must be made more accessible and affordable for those who benefit from them. With regards to NTDs, this requires more accurate collection and reporting of data in terms of areas in need of surgical care, as well as an adequate scale-up of surgical services in these areas. LF and trachoma are 2 examples of NTDs with surgical comorbidities that make up a significant portion of the disease burden, the elimination of which will also require mass-scale surgical management.

### Trachoma

Trachoma is an ocular bacterial infection that results in inflammation of the conjunctiva, “active trachoma.” Repeated bouts of active trachoma can scar the inner eyelid, which often leads to trichiasis—an inward turning of 1 or more eyelashes, damaging the cornea [6]. This is an extremely painful condition that will lead to irreversible blindness if not surgically corrected in a timely manner. There are over 200 million people living in areas with a high risk of trachoma-related blindness [6]. The recommended procedure for trichiasis is known as bilamellar tarsal rotation (BLTR) surgery [6]. BLTR surgery carries a relatively small estimated cost of US$40 and remains a cost-effective intervention against vision loss [7].

Unlike with most other NTDs, surgical interventions in trachoma have been an integral part of the framework, supported by the World Health Organization (WHO) Alliance for Global Elimination of Blinding Trachoma by the year 2020 (GET 2020) [6]. They have been implemented globally as SAFE: surgery for trachomatous trichiasis (TT); antibiotic treatment to clear ocular infection; facial cleanliness to reduce the transmission of ocular *Chlamydia trachomatis*; and environmental improvement, particularly access to water and sanitation [6].

A recent achievement toward the goal of elimination was the Global Trachoma Mapping Project (GTMP), which has identified 100 million additional individuals living in areas previously known as “suspected endemic” that will require health interventions [5]. As shown by this project, the proper identification of areas that require intervention is a prerequisite for the achievement of the true elimination of any NTD [5]. In 2012, 169,121 people received trichiasis surgery, and in 2013, that number increased to 234,000 [6]. However, there is currently an estimated backlog of 5 million cases of trichiasis surgery [8]. Thus, the current level of productivity is not adequate to reach the GET 2020 targets, and there is a clear need to scale up trichiasis surgery services globally [9].

### LF

LF is a vector-borne parasitic disease that arises from the infection of lymphatic vessels with adult filarial worms, eventually leading to chronic disabling sequelae, including hydrocele, lymphedema, and elephantiasis [10]. Of these, hydrocele is curable through surgery (hydrocelectomy), while lymphedema and elephantiasis can be managed with nonsurgical techniques, such as improved hygiene and skin care [11]. MDA treatments function to decrease the prevalence of infection by lowering the concentration of microfilariae in the blood to a point at which transmission is no longer possible [10, 11]. Thus, MDA is a major tool in the global elimination of LF, but it does not significantly address hydroceles requiring surgical interventions or lymphedema and elephantiasis morbidities that would be addressed through improved hygiene, compression, and antibiotics.

Over the past 15 years (2000–2015), since the inception of the Global Programme to Eliminate Lymphatic Filariasis (GPELF), more than 97 million estimated cases of LF have been prevented or cured predominantly through MDA [10]. However, as many as 36 million hydrocele and lymphedema cases remain [10]. More recent Global Burden of Disease Study 2015 estimates show that, for the 38.46 million people living with LF, approximately 19.71 million show evidence of detectable microfilariae, while 18.76 million exhibit complications that could require surgical intervention (Table 1) [12].

**Table 1:** Global morbidities due to LF and trachoma (from the GBD 2015 [12]).

Disease	Prevalence in 2015	YLDs in 2015
Trachoma	3.56 million	279,200
LF–prevalence of detectable microfilaria	19.71 million	0
LF–Complications aggregate	18.76 million	2.075 million
Total	42.03 million	2.354 million

Abbreviations: GBD, Global Burden of Disease Study; LF, lymphatic filariasis; YLDs, years lost from disability.

For the GPELF to reach its goal, patient care—as achieved through the implementation of morbidity management and surgery—is needed in conjunction with MDA, given that there are a large number of people with existing severe disease for whom MDA would prove too late to be effective [8]. Preventing the further progression of disease in these patients requires morbidity management and disability prevention (MMDP) services. Surgery is an essential part of MMDP, as seen in the minimum package of recommended care suggested by WHO for the care of patients with LF-related chronic disease. The minimum package includes hydrocelectomy, treatment for adenolymphangitis (ADL), and lymphedema management in order to prevent ADL episodes and subsequent disease progression [10]. Hydrocele surgery is estimated at US$80–US$360, and the cost of procedures for lymphedema prevention and management is even smaller [7].

In addition to the health burden, people suffering from chronic LF complications could incur losses of up to 30% in productivity, translating to the economic loss of hundreds of millions of dollars each year [13,14,15]. Thus, MMDP must be implemented in conjunction with MDA to achieve the elimination of LF as a health and economic burden, and surgical care is a crucial part of this effort.

## What needs to happen?

### Trachoma

In order to increase productivity and clear the existing backlog in trichiasis surgery cases, a significant scale-up and refocusing of surgical services will be necessary. Specifically, with regards to the backlog, it may be necessary to institute urgent responses that address a large number of cases at one time (campaign-style approach) [9]. In addition, matching resource allocation will be required, and a refocusing of surgical resources to areas where patients are most concentrated would serve to improve productivity. Some of the barriers to low uptake that have been identified are lack of awareness, direct and indirect costs, and distance to services [9]. Thus, local promotion strategies, such as using village leaders to encourage patients to seek care at health facilities, have been proposed as strategies to increase surgical access [9]. Other studies have also observed that a recurrence of trichiasis following surgery is relatively common and has been frequently tied to surgeon skill [16]. Proper standardization and supervision of procedures could reduce adverse outcomes and improve surgeon skill. Additionally, 1 dose of azithromycin given postoperatively has been shown to yield a 30% reduction in recurrence in Ethiopia [17].

### LF

Many of the strategies in the surgical framework utilized for trachoma elimination efforts can be reasonably extrapolated to expand surgical coverage for hydrocele repair. For example, the mapping of LF, and specifically hydrocele, prevalence in subregions within endemic areas by using rapid antigen testing, along with mobile technology (as seen in GTMP), could prove useful in identifying local regions in need of access to surgical care [8]. The involvement of members at the rural level who can help identify and promote individuals to seek proper surgical care in a timely manner could also aid in surgical uptake [9]. Additionally, as shown in a recent study in Malawi, better characterization of regional surgical output and access within endemic countries and geographical distribution of hydrocele patients in relation to the servicing facility could improve the monitoring of progress and guide future scale-up of services [18].

Currently, of the 73 LF-endemic countries, only 41 (56%) have reported lymphedema cases, and 38 (52%) have done so for hydrocele cases [10]. Of those, 35 countries (48%) reportedly provided MMDP services, but only a mere 18 countries (25%) have shown evidence of the surveillance of MMDP at the level of the implementation unit [10]. Six of the 18 endemic countries conducting surveillance have thus far been assessed and formally acknowledged as having achieved elimination [10]. However, the goal, as set by WHO, is for 100% of endemic countries to enter postintervention surveillance by 2020 [10]. Needless to say, this will require a large scale-up and expansion of surgical care, along with other MMDP services.

## Who will do it?

One of the biggest challenges regarding access to surgical services is the issue of who, exactly, will be performing these procedures at the regional level. In addressing this issue, the mission should be 2-fold: to clear the backlog of existing unoperated cases efficiently and to provide a sustainable system at existing facilities that maintain ongoing care.

The unassailable primary players in the realm of global surgery are the surgeons, whether native to the region or visiting. As previously mentioned, one of the strategies that has been proposed to clear the backlog in the case of trichiasis surgery is the campaign-style approach. There is evidence to suggest that well-planned and effectively executed campaigns can provide as many as 20 trichiasis surgeries per surgeon per day [9].

This idea of mass surgery camps has also been experimented with in the case of hydrocele repair for LF. A Nigerian study found that outreach via mass surgery camps at district hospitals performed by qualified surgeons had low postoperative complication rates and contributed in large part to decreasing overall disease burden [19]. However, in order to achieve adequate scale-up of services long term in either case, there must be a form of an established ongoing system of surgical care in between outreach camps in order to completely address the disease burden, particularly focusing on recurrent and incident cases.

Considering that in most countries there are too few trained surgeons to address the current disease burden, it is also important to recruit existing local healthcare workers such as general practitioners or associate clinicians to provide surgical care. This is a process called task shifting, as discussed in the commission’s report, in which dedicated healthcare workers could be trained separately to perform specific simple procedures, such as trichiasis surgery or hydrocele repair [2]. Ultimately, both LF and trachoma surgeries need to be incorporated into national health systems and into programs for the strengthening of health systems in areas where this is not already occurring.

In the case of trachoma, trained eye care workers have already been employed to properly diagnose trichiasis and provide surgeon referral for cases requiring surgery. Further training these specialized eye care workers to perform simple procedures such as trichiasis surgery could go a long way in helping scale up surgical services [9]. BLTR surgeries performed by integrated eye care workers have been shown to yield similar outcomes to those performed by ophthalmologists [20]. With LF, WHO recommendations state that a standard, uncomplicated hydrocelectomy can be routinely performed in peripheral (primary) health facilities [21].

Similar research has been done in using community health workers in tackling local disease burden in other NTDs, including leprosy [22]. Thus, there must be a push toward the allocation of resources toward specialized training of primary healthcare workers for simple procedures and recognition of a cadre of specialist healthcare workers by the ministries of health with regards to NTDs, primarily LF and trachoma.

## Concluding statement

Together, the chronic complications resulting from LF and trachoma result in more than 2.3 million years lost from disability (YLDs) [12]. Therefore, in addition to supporting global MDA efforts, it is imperative that the current major donors—currently the United States and the United Kingdom—continue to provide support for comorbidity management. According to the US Agency for International Development (USAID) and their NTD program, they currently “also provide support to countries to assist with morbidity management and disability prevention, with a focus on high-quality treatment and care” [23].

As the evidence of the importance of scaling up surgical services in the road to eradicating NTDs increases, it is crucial that these organizations continue to expand funding and allocate funds specifically toward increasing access to surgical care.

Ultimately, the role of the G7 countries, as well as the ministries of health in endemic countries, will be paramount in the focus toward scaling up surgical services within disease-endemic countries. However, based on studies showing that most of the world’s LF cases are found in comparatively wealthy G20 nations and Nigeria, they must also be encouraged to tackle their own NTD issues and expand domestic efforts [24,25].

Trachoma and LF surgical comorbidities represent important and salient challenges to the Lancet Commission on Global Surgery and their international stakeholders. Providing global access to the simple surgeries required for these NTDs represents a key first step toward universal surgical access.

## References

[1] Meara JG, Hagander L, Leather AJ. Surgery and global health: a Lancet Commission. Lancet. 2014;383(9911):12–3. doi: 10.1016/S0140-6736(13)62345-4 24332309 10.1016/S0140-6736(13)62345-4

[2] Meara JG, Leather AJ, Hagander L. Global Surgery 2030: evidence and solutions for achieving health, welfare, and economic development. Lancet. 2015;386:569–624. doi: 10.1016/S0140-6736(15)60160-X 25924834 10.1016/S0140-6736(15)60160-X

[3] The Global Network for Neglected Tropical Diseases. Neglected Tropical Diseases and the Post-2015 Development Agenda. Available from: http://www.sabin.org/sites/sabin.org/files/resources/final_post-2015_brief_update_11.7.2014.pdf. [cited 2017 Jan 7].

[4] Webster JP, Molyneux DH, Hotez PJ, et. al The contribution of mass drug administration to global health: past, present and future. Phil. Trans. R. Soc. B. 2014;369:20130434. 10.1098/rstb.2013.0434 PMC4024227 24821920

[5] Reaching The Unreached: Fourth progress report of the London Declaration. Available from: http://unitingtocombatntds.org/sites/all/themes/f1ux/images/fourth-report/en/pdfs/4th-Report-on-Progress_EN.pdf. [cited 2017 Jan 7].

[6] World Health Organization. WHO Alliance for the Global Elimination of Blinding Trachoma by the year 2020. WHO Weekly Epidemiological Record. 2014;89(39):421428. 25275153

[7] World Health Organization. Investing to overcome the global impact of neglected tropical diseases: Third WHO report on neglected tropical diseases. 2015.

[8] Molyneux DH, Savioli L, Engels D. Neglected tropical diseases: progress towards addressing the chronic pandemic. Lancet. 2016;6736(16):30171–4. 10.1016/S0140-6736(16)30171-4 27639954

[9] Global Scientific Meeting on Trachomatous Trichiasis. Available from: http://www.trachomacoalition.org/sites/default/files/content/resources/files/Moshi_TTSWR_English_2013.4.19.pdf. [cited 2017 Jan 7].

[10] World Health Organization. Global Programme to Eliminate Lymphatic Filariasis: Progress Report, 2015. WHO Weekly Epidemiological Record. 2016;91(39):441–460.

[11] World Health Organization. Bridging the gap World Health Report. World Health Organization, Geneva, Switzerland 1995.

[12] GBD 2015 Disease and Injury Incidence and Prevalence Collaborators. Global, regional, and national incidence, prevalence and years lived with disability for 310 diseases and injuries, 1990–2015: a systematic analysis for the Global Burden of Disease Study 2015. Lancet. 2016;388:1545–602. doi: 10.1016/S0140-6736(16)31678-6 27733282 10.1016/S0140-6736(16)31678-6 PMC5055577

[13] Ramaiah KD, Das PK, Michael E, et. al The economic burden of lymphatic filariasis in India. Parasitology Today. 2000;16(6):251–253. 10827432 10.1016/s0169-4758(00)01643-4

[14] Managing morbidity and preventing disability in the Global Programme to Eliminate Lymphatic Filariasis: WHO position statement. Geneva, World Health Organization; 2011 (WHO/HTM/NTD/PCT/2011.8). 22191103

[15] Addiss DG, Brady MA. Morbidity management in the Global Programme to Eliminate Lymphatic Filariasis: a review of the scientific literature. Filaria Journal. 2007;6:2 doi: 10.1186/1475-2883-6-2 17302976 10.1186/1475-2883-6-2 PMC1828725

[16] Solomon A. Optimising the management of trachomatous trichiasis Lancet Glob Health. 2016;4:e140–41. doi: 10.1016/S2214-109X(16)00004-8 26774709 10.1016/S2214-109X(16)00004-8 PMC7116499

[17] West SK. Triachisis Recurrence—Why the Surgery Part of Trachoma Control is Still Uncontrolled. US Ophthalmic Review. 2011;4(1):77–9

[18] Stanton MC, Smith EL, Martindale S, et. al Exploring hydrocele surgery accessibility and impact in a lymphatic filariasis endemic area of southern Malawi. Trans R Soc Trop Med Hyg. 2015;109:252–261. doi: 10.1093/trstmh/trv009 25673628 10.1093/trstmh/trv009

[19] Thomas G, Richards FO Jr, Eigege A et al A pilot program of mass surgery weeks for treatment of hydrocele due to lymphatic filariasis in central Nigeria. Am J Trop Med Hyg. 2009;80:447–51. 19270297

[20] Alemayehu W, Melese M, Bejiga A, et al Surgery for trichiasis by ophthalmologists versus integrated eye care workers: a randomized trial. Ophthalmology. 2004;111(3):57884. 10.1016/j.ophtha.2003.06.030 15019339

[21] World Health Organization. Lymphatic filariasis: Managing morbidity and preventing disability. Geneva, World Health Organization 2013 Available from: http://apps.who.int/iris/bitstream/10665/85347/1/9789241505291_eng.pdf. [cited 2017 Jan 7].

[22] Corley AG, Clifton PT, Glass NE. The Role of Nurses and Community Health Workers in Confronting Neglected Tropical Diseases in Sub-Saharan Africa: A Systematic Review. PLoS Negl Trop Dis. 2016;10(9):e0004914 doi: 10.1371/journal.pntd.0004914 27631980 10.1371/journal.pntd.0004914 PMC5025105

[23] USAID National Tropical Diseases Program: Lymphatic Filariasis. Available from: https://www.neglecteddiseases.gov/usaid-target-diseases/lymphatic-filariasis. [cited 2017 Jan 9].

[24] Hotez PJ. NTDs V.2.0: ‘Blue Marble Health’: neglected tropical disease control and elimination in a shifting health policy landscape. PLoS Negl Trop Dis. 2013;7:e2570 doi: 10.1371/journal.pntd.0002570 24278496 10.1371/journal.pntd.0002570 PMC3836998

[25] Hotez PJ, Damania A, Naghavi M. Paying for Worms. PLoS Negl Trop Dis. 2016;10(12):e0005092 doi: 10.1371/journal.pntd.0005092 28033369 10.1371/journal.pntd.0005092 PMC5199027

